# Testing the Intercept of a Balanced Predictive Regression Model

**DOI:** 10.3390/e24111594

**Published:** 2022-11-02

**Authors:** Qijun Wang, Xiaohui Liu, Yawen Fan, Ling Peng

**Affiliations:** 1 School of Statistics, Jiangxi University of Finance and Economics, Nanchang 330013, China; 2 Key Laboratory of Data Science in Finance and Economics, Jiangxi University of Finance and Economics, Nanchang 330013, China

**Keywords:** balanced predictive regression model, intercept, empirical likelihood, stationary, non-stationary, C12, C22

## Abstract

Testing predictability is known to be an important issue for the balanced predictive regression model. Some unified testing statistics of desirable properties have been proposed, though their validity depends on a predefined assumption regarding whether or not an intercept term nevertheless exists. In fact, most financial data have endogenous or heteroscedasticity structure, and the existing intercept term test does not perform well in these cases. In this paper, we consider the testing for the intercept of the balanced predictive regression model. An empirical likelihood based testing statistic is developed, and its limit distribution is also derived under some mild conditions. We also provide some simulations and a real application to illustrate its merits in terms of both size and power properties.

## 1. Introduction

Prediction of some important variables based on the other available predictors is a practice of great interest in many applications in finance and economics. Various predictive regression models have been suggested in the last few decades. Among them, the simplest one is the following linear predictive regression model:

(1) $$\begin{array}{c} \left\{\begin{array}{c} Y_{t}=\alpha +\beta X_{t-1}+U_{t}\\ X_{t}=\mu +\phi X_{t-1}+e_{t},\;B\left(L\right)e_{t}=V_{t}, \end{array}\right. \end{array}$$

where α and β are unknown parameters, $Y_{t}$ denotes the response, and $X_{t}$ the predictor. In practice, $Y_{t}$ may denote the stock return, and $X_{t}$ may be some financial indicators. $L^{i}e_{t}=e_{t-i}$, $B\left(L\right)=1-\left(\sum_{i=1}^{p}b_{i}L^{i}\right)$, B(L)≠0 with all of its roots are fixed and less than one in absolute value. ${\left\{U_{t},V_{t}\right\}}_{t=1}^{n}$ are independent and identically distributed (hereafter i.i.d.) random vectors with zero mean. For the predictor $X_{t}$, μ denotes the intercept term, and ϕ is the autoregressive coefficient. It is known that the limit distribution of the least squares estimators of α and β differs from each other when $\left\{X_{t}\right\}$ follows: (i) |ϕ|<1 (stationary), (ii) μ=0 and ϕ=1+c/n for some constant *c*, (iii) μ≠0 and ϕ=1+c/n for some constant *c*. When c=0, $\left\{X_{t}\right\}$ is a unit root AR(1) process.

Predictive regression models are widely used in financial research (see [1,2,3,4,5,6,7,8], and references therein). It is noted that, when the predictor $\left\{X_{t}\right\}$ is non-stationary, the response $\left\{Y_{t}\right\}$ can not be stationary. This is well known in the literature as the unbalanced problem for traditional predictive regression models. Due to the non-stationarity of most financial variables (see [9]), the problem of unbalance often appears in practical applications. To cover this problem, Ref. [10] proposed a balanced predictive regression (BPR) model by adding a difference term of $X_{t}$ to the conditional function part. In detail, it takes the form as follows:

(2) $$\begin{array}{c} \left\{\begin{array}{c} Y_{t}=\alpha +\beta X_{t-1}+\sum_{i=1}^{p}\psi_{i}\Delta X_{t-i}+U_{t}\\ X_{t}=\mu +\phi X_{t-1}+e_{t},\;B\left(L\right)e_{t}=V_{t}, \end{array}\right. \end{array}$$

where $\Delta X_{t-i}=X_{t-i}-X_{t-i-1}$, and $\psi_{i}$’s are unknown parameters needing to be estimated. Compared to the original predictive regression model (1), the existence of $\Delta X_{t-i}$’s in (2) can balance the unmatched problem between $Y_{t}$ and $X_{t}$ with β=0 when $\left\{X_{t}\right\}$ is non-stationary.

Similar practices are also considered by [11], which proposes a balanced predictive regression model by adding an additional lag of the predictors to the mean function. Through simple transformation, this model is the same as that considered in this paper when p=1 by noting that $\beta X_{t-1}+\psi_{1}\Delta X_{t-1}=\left(\beta +\psi_{1}\right)X_{t-1}+\left(-\psi_{1}\right)X_{t-2}$. Both for predictive regression and BPR models, an importation issue of great interest is to test the predictability, i.e., testing the null hypothesis $H_{0}:\beta =0$ or $H_{0}:\psi_{1}=\cdots =\psi_{p}=0$ or $H_{0}:\beta =\psi_{1}=\cdots =\psi_{p}=0$. This issue has received extensive attention in the literature. The conventional method for testing predictability is the so-called *t*-test. Unfortunately, the standard *t*-test suffers from the issue of obvious over-rejection or ineffective when predictors have strong persistence. See [12,13] for more details. Note that [14] investigated the estimation of predictive regressions when the explanatory variable is nearly integrated. However, the limiting distributions of these tests depend on which **Case** of **(i)–(iii)** the predictor $X_{t}$ follows. Some unified predictability tests have been developed in the last decade to avoid this problem. Among others, Ref. [15] considered the predictability test for model (1) when macroeconomic data are non-stationary or heavy-tailed. For the BPR model, Ref. [10] extended the method of [15] and proposed a unified predictability test regardless of $X_{t}$ being stationary, nearly integrated, or unit root.

However, two statistics were developed in [10] to test predictability, which depends on a predefined assumption on whether or not there exists an intercept term α. Although both testing statistics are shown to be chi-squared distributed asymptotically under mild conditions, their finite sample performances are quite different from each other because, when there is an intercept, their second testing statistic depends on the idea of data splitting in order to get rid of the effect of the existence of the intercept term α. Note that both tests are applicable when the underlying true model has no intercept. We found that the *p*-values of these two tests are different, which may cause confusion when different *p*-values are obtained based on the same data set.

Hence, it is necessary to do the predefined test to check whether or not there exists a non-zero intercept in model (2) before conducting a predictability test. This motivates us to consider the current research. We propose an empirical likelihood test based on the idea of data splitting and proved that the asymptotic distribution of the test statistic is $\chi^{2}$ distribution, regardless of $X_{t}$ being stationary or nearly integrated or unit root. The empirical likelihood method was proposed by [16] and proved to have many excellent properties [17], including the distribution of the data that does not need to be assumed. Therefore, this method has been widely studied in the literature [18,19,20]. This also prompted this paper to propose a unified test of interception terms using the empirical likelihood method.

The rest of this article is organized as follows: Section 2 presents the methodologies and the main asymptotic results of our proposed test. Section 3 contains the finite sample simulation studies. An empirical application is discussed in Section 4. The detailed proofs of the main results are presented in Appendix A.

## 2. Methodology and Main Results

Suppose the random observations ${\left\{X_{t},Y_{t}\right\}}_{t=1}^{n}$ are generated from the model (2). The main interest of this paper is to develop a unified test for checking the intercept for model (2), i.e., the following hypothesis

$$H_{0}:\alpha =\alpha_{0},\;\mathrm{versus}\;H_{1}:\alpha \neq \alpha_{0}.$$

Hereafter, denote $\theta_{0}:={\left(\alpha_{0},\beta_{0},\psi_{1,0},\cdots ,\psi_{p,0}\right)}^{⊤}$ as the true value of $\theta :={\left(\alpha ,\beta ,\psi_{1},\cdots ,\psi_{p}\right)}^{⊤}$ without confusion.

Since the empirical likelihood method enjoys many desirable properties as discussed in the literature, in the sequel, we are interested in developing a testing method for $H_{0}$ based on this method. Note that, when $\theta =\theta_{0}$, we have

$$\begin{array}{c} E\left(\left(Y_{t}-\alpha -\beta X_{t-1}-\sum_{i=1}^{p}\psi_{i}\Delta X_{t-i}\right)\xi_{t}\right)=0, \end{array}$$

where $\xi_{t}:={\left(1,X_{t-1},\Delta X_{t-1},\cdots ,\Delta X_{t-p}\right)}^{⊤}$. Then, following [16], one may construct an empirical likelihood function by using the auxiliary vectors $\left\{W_{t}\right\}$, where

$$W_{t}\left(\theta \right):=\left(Y_{t}-\alpha -\beta X_{t-1}-\sum_{i=1}^{p}\psi_{i}\Delta X_{t-i}\right)\xi_{t}.$$

Unfortunately, this test will suffer from the trouble of the quantity $\frac{1}{n}\sum_{t=p+1}^{n}\{D_{n}^{-1}$$W_{t}\left(\theta \right)W_{t}{\left(\theta \right)}^{⊤}D_{n}^{-1}\}$ not converging in probability as n→∞ for **Case (i)**, i.e., with μ=0 and ϕ=1+c/n for some constant *c*, where $D_{n}=\mathrm{diag}\{1,\sqrt{n},1$, ⋯,1}. Consequently, the quantity

$$\begin{array}{c} \left(\frac{1}{\sqrt{n}}\sum_{t=p+1}^{n}D_{n}^{-1}W_{t}\left(\theta \right)\right){\left(\frac{1}{n}\sum_{t=p+1}^{n}D_{n}^{-1}W_{t}\left(\theta \right)W_{t}{\left(\theta \right)}^{⊤}D_{n}^{-1}\right)}^{-1}\left(\frac{1}{\sqrt{n}}\sum_{t=p+1}^{n}D_{n}^{-1}W_{t}\left(\theta \right)\right) \end{array}$$

does not converge in distribution to a chi-squared distributed variable, which in turn results in the related log-empirical likelihood function having a non-standard limit distribution.

As an improvement, although it is possible to construct a new empirical likelihood-based test statistic, which converges in a chi-squared distribution, for θ relying on the idea of data splitting as did in [21], the main interest of this paper is to test the intercept term, i.e., hypothesis $H_{0}$, and we need to handle the redundant parameters $\beta ,\psi_{1},\cdots ,\psi_{p}$ by using the profile empirical likelihood method as in [17]. Once again, this profile method still does not work; see Theorem 3 in [21] for a detailed discussion.

This motivates us to consider the following testing procedure. Let $\psi ={\left(\psi_{1},\cdots ,\psi_{p}\right)}^{⊤}$, $Z_{t}\left(\alpha ,\beta ,\psi \right)={\left(Z_{t,1}\left(\alpha ,\beta ,\psi \right),Z_{t,2}\left(\alpha ,\beta ,\psi \right),\cdots ,Z_{t,2+p}\left(\alpha ,\beta ,\psi \right)\right)}^{⊤}$ for t=p+1,2,⋯,m, where m=[n/2] with [·] being the floor function, and

$$\begin{array}{c} \left\{\begin{array}{c} Z_{t,1}\left(\alpha ,\beta ,\psi \right)=Y_{t}-\alpha -\beta X_{t-1}-\sum_{i=1}^{p}\psi_{i}\Delta X_{t-i}\\ Z_{t,2}\left(\alpha ,\beta ,\psi \right)=\left(Y_{t+m}-\alpha -\beta X_{t+m-1}-\sum_{i=1}^{p}\psi_{i}\Delta X_{t+m-i}\right)w_{t+m}+{\bar{\delta }}_{t}\\ Z_{t,2+k}\left(\alpha ,\beta ,\psi \right)=\left(Y_{t}-\alpha -\beta X_{t-1}-\sum_{i=1}^{p}\psi_{i}\Delta X_{t-i}\right)\Delta X_{t-k},\;k=1,2,\cdots ,p, \end{array}\right. \end{array}$$

with $w_{t}=\frac{X_{t-1}}{\sqrt{1+X_{t-1}^{2}}\log(e+|X_{t-1}\left|\right)}$, and ${\bar{\delta }}_{t}=\frac{1}{\sqrt{N}}\sum_{i=1}^{N}\delta_{i,t}$ with N=2000 and $\delta_{i,t}∼N\left(0,{\bar{\sigma }}^{2}\right)$, which are independent of the random observations. Through data splitting technology, we use the first half of the data to estimate redundant parameters, and the second half of the data to construct a likelihood function to reduce the challenges posed by noise in the data. To balance the size and the power, in practice, one may take ${\bar{\sigma }}^{2}={\hat{\sigma }}_{U}^{2}/2$ with ${\hat{\sigma }}_{U}^{2}$ being the estimated error variance. Note that here $\log(e+|X_{t-1}|)$ is designed in order to let the quantity $\frac{1}{m}\sum_{t=p+1}^{m}\left\{\left(Y_{t+m}-\alpha -\beta X_{t+m-1}-\sum_{i=1}^{p}\psi_{i}\Delta X_{t+m-i}\right)w_{t+m}\right\}$ to vanish when $\left\{X_{t}\right\}$ follows the non-stationary **Cases (ii)**. A similar weighting technique has also been used in [22]. which uses the profile empirical likelihood to consider the intercept test of the predictive regression model.

Based on $\left\{Z_{t}\left(\alpha ,\beta ,\psi \right)\right\}$ above, a new empirical likelihood function for θ can be defined as follows:

$$\begin{array}{c} L\left(\alpha ,\beta ,\psi \right)=\sup\left\{\prod_{t=p+1}^{m}\left(mp_{t}\right):p_{1}\geq 0,\cdots ,p_{m}\geq 0,\;\sum_{t=p+1}^{m}p_{t}=1,\;\sum_{t=p+1}^{m}p_{t}Z_{t}\left(\alpha ,\beta ,\psi \right)=0\right\}. \end{array}$$

Since we are only interested in α, we further define a profile empirical likelihood function for α as

$$\begin{array}{c} \ell \left(\alpha \right)=\underset{\left(\beta ,\psi \right)}{\sup}L\left(\alpha ,\beta ,\psi \right). \end{array}$$

The following theorem shows that Wilks’ theorem holds for the above proposed profile empirical likelihood method, which depends on some regular conditions as follows:

(C1) $\left\{\left(U_{t},e_{t}\right)\right\}$ is a sequence of i.i.d. random vectors with mean zero, and $E(|U_{t}{|}^{2+\epsilon_{0}})+E(|e_{t}{|}^{2+\epsilon_{0}})<\infty$ for some arbitrarily small positive constant $\epsilon_{0}$;
(C2) The predictive variable $X_{t}$, with the initial value of $X_{0}$ being constant or random variable of order $o_{p}\left(\sqrt{m}\right)$, belongs to one of the persistence classes following **Cases (i)–(iii)**;
(C3) All roots of $1-\sum_{i=1}^{p}\psi_{i,0}x^{i}=0$ with respect to *x* are outside the unit circle.

**Theorem** **1.** *Suppose that Conditions C1–C3 hold. Then, under the null hypothesis $H_{0}:\alpha =\alpha_{0}$, we have that $-2\log\ell (\alpha_{0})$ converges in distribution to a chi-squared distributed variable $\chi_{1}^{2}$ with one degree of freedom as n→∞.*

Based on the above theorem, we can reject $H_{0}$ once

$$-2\log\ell \left(\alpha_{0}\right)\geq \chi_{1}^{2}\left(1-\tau \right),$$

at the significance level τ∈(0,1), where $\chi_{1}^{2}\left(1-\tau \right)$ denotes the (1−τ)-th quantile of a chi-squared distribution with one degree of freedom. In particular, when one is interested in testing $H_{0}:\alpha =0$, one may think that there is no intercept in model (2) if $-2\log\ell \left(0\right)\geq \chi_{1}^{2}\left(1-\tau \right)$ at the significance level τ.

Note that, similar to [23], one may take $w_{t}=\frac{X_{t-1}}{{\left(1+X_{t-1}^{2}\right)}^{0.75}}$ to construct the test. Here, we however use an another weight $w_{t}=\frac{X_{t-1}}{\sqrt{1+X_{t-1}^{2}}\log(e+|X_{t-1}\left|\right)}$ in $Z_{t,2}\left(\alpha ,\beta ,\psi \right)$ in order to increase the local power of the proposed test for the non-stationary cases. The following theorem states the power property of our proposed empirical likelihood-based test.

**Theorem** **2.** *Suppose the same conditions of Theorem 1 hold. Then, we have as* n→∞ *that:*

- *For **Case (i)**, under the local alternative hypothesis $H_{1}:\alpha =\alpha_{0}-\frac{d_{0}}{\sqrt{m}}$ for some constant $d_{0}\in R$,*
$$-2\log\ell \left(\alpha_{0}\right)\overset{d}{⟶}\chi_{1}^{2}\left(\nu_{1}^{2}\right),$$
  *where ‘$\overset{d}{⟶}$’ denotes the convergence in distribution, and $\chi_{1}^{2}\left(\nu_{1}^{2}\right)$ a non-central chi-squared distributed variable with non-central parameter $\nu_{1}^{2}$, where $\nu_{1}$ is the second component of $\Sigma^{-1/2}\gamma_{1}$ with $\gamma_{1}=d_{0}{\left(1,\lim_{t\to \infty }E\left(w_{t+m}\right),\mu +\left(\phi -1\right)\lim_{t\to \infty }E\left(X_{t-2}\right),\cdots ,\mu +\left(\phi -1\right)\lim_{t\to \infty }E\left(X_{t-p-1}\right)\right)}^{⊤}$, and*
$$\begin{array}{c} \Sigma =\lim_{t\to \infty }E\left(Z_{t}\left(\alpha_{0},\beta_{0},\psi_{0}\right)Z_{t}{\left(\alpha_{0},\beta_{0},\psi_{0}\right)}^{⊤}\right); \end{array}$$
- *For **Case (ii)**, under the local alternative hypothesis $H_{1}:\alpha =\alpha_{0}-\frac{d_{0}\log\left(m\right)}{\sqrt{m}}$ for some constant $d_{0}$,*
$$\begin{array}{c} -2\log\ell \left(\alpha_{0}\right)\overset{d}{⟶}{\left(\xi +\frac{2d_{0}}{\bar{\sigma }}\int_{1}^{2}sgn\left(J_{c}\left(s\right)\right)ds\right)}^{2}, \end{array}$$
  *where sgn(·) denotes the sign function, ξ a standard normally distributed variable, which is independent of $\int_{1}^{2}sgn\left(J_{c}\left(s\right)\right)ds$, and $J_{c}\left(s\right):=\int_{0}^{s}e^{-(s-r)c}dW\left(r\right)$ with W(r) being a Gaussian process with covariance function $\left(s,t\right)=2E\left(e_{t}^{2}\right)\min\left(s,t\right)$;*
- *For* ***Case (iii)*** *, under the local alternative hypothesis $H_{1}:\alpha =\alpha_{0}-\frac{d_{0}\log\left(m\right)}{\sqrt{m}}$ for some constant $d_{0}$,*
$$\begin{array}{c} -2\log\ell \left(\alpha_{0}\right)\overset{d}{⟶}\chi_{1}^{2}\left(\nu_{2}^{2}\right), \end{array}$$
  *where $\nu_{2}=d_{2}\int_{1}^{2}sgn\left(\mu \int_{0}^{s}e^{-(s-r)c}dr\right)ds/\bar{\sigma }$.*

Theorem 2 indicates that the local power of the proposed test for **Cases (ii)** and **(iii)** is of order $O(\frac{\log(m)}{\sqrt{m}})$, which does not achieve the normally parametric order $O(1/\sqrt{m})$ but is better than $O(n^{-a})$ for any a∈(0,1/2).

**Remark** **1.** *Although we only consider in Theorems 1 and 2 the case that $\left\{U_{t}\right\}$ is a sequence of i.i.d. errors, it is possible to obtain similar results when $U_{t}$ follows a dependent process. In fact, when $U_{t}$ follows the AR(q) process, one can obtain similar limits as in Theorems 1 and 2 by taking into account the AR structure of $U_{t}$.*

**Remark** **2.** *When $e_{t}$ follows a strictly stationary GARCH process or a linear process, Theorems 1 and 2 still hold.*

## 3. Simulation Results

In this section, we investigate the finite sample performance of the proposed profile empirical likelihood test using simulated data sets and compare it with the traditional *t*-test. The random observations $\left\{X_{t},Y_{t}\right\}$ are generated from the predictive regression model (2), where α=0, $\beta =\left\{\beta_{0},\beta_{1}\right\}=\left\{0.5,0.3\right\}$. μ∈{0,0.01} indicates that the model has no intercept item and intercept item, respectively. $\phi \in \{0.5,1,1-\frac{3}{n}\}$, where 0.5 indicates that $X_{t}$ is a stationary process, 1 indicates a unit root process, and $1-\frac{3}{n}$ indicates a near unit root process. The innovations ${\left\{U_{t},V_{t}\right\}}_{t=1}^{n}$ were considered in two cases.

**Case (i)**: ${\left\{U_{t},V_{t}\right\}}_{t=1}^{n}$ are from a bivariate Gaussian Copula $C(F_{1}\left(U_{t}\right),F_{2}\left(V_{t}\right),\rho )$, whose marginal distribution $F_{i}$ is a Student’s *t*-distribution with degrees of freedom df∈{10,15}. The dependence parameter ρ is set to be {−0.2, −0.4, −0.6}, respectively, to explore the performance of two innovations under different degrees of dependence.

**Case (ii)**: ${\left\{V_{t}\right\}}_{t=1}^{n}$ are from GARCH(1,1) structure, i.e.,

$$\left\{\begin{array}{c} V_{t}=\eta_{v,t}\sigma_{v,t},\\ \sigma_{v,t}^{2}=\omega_{v}+a_{v}V_{t-1}^{2}+b_{v}\sigma_{v,t-1}^{2}. \end{array}\right.$$

where $\left\{\omega_{v},a_{v},b_{v}\right\}=\left\{0.1,0.4,0.2\right\}$. ${\left\{U_{t}\right\}}_{t=1}^{n}$ and ${\left\{\eta_{v,t}\right\}}_{t=1}^{n}$ are generated similarly to ${\left\{U_{t},V_{t}\right\}}_{t=1}^{n}$ in **Case (i)**. All simulating results are repeatedly carried 10,000 times with the sample size *n* ranging from 200 to 1200.

Table 1 reports the size performance of the proposed method with different settings in **Case (i)** under the significance level τ=0.05. For df=(10,10), the results show that, when $X_{t}$ is a stationary or unit root process, regardless of whether μ is 0 or not, with the increase of sample *n*, size values are closer to τ, and the performance of the method is robust under different ρ. However, when $X_{t}$ is a near unit root process, it can be found that, when *n* is small, the method has the performance of oversize, especially when ρ=−0.6, and as *n* increases, it gradually converges to the significance level. For the *t*-test when $X_{t}$ is a stable process, the *t*-test has a good size performance, and its performance is relatively stable even with high dependency. However, when $X_{t}$ is a unit root or near unit root process, it gradually shows over-rejecting with the increase of ρ, no matter whether there is an intercept or not. The df=(15,15) behaves similarly to df=(10,10). Table 2 reports the size performance in **Case (ii)**, in which the innovation of $X_{t}$ has a GARCH structure. The results show that the performances of the profile empirical likelihood and *t*-test are similar to that in **Case (i)**.

**Figure 1:**
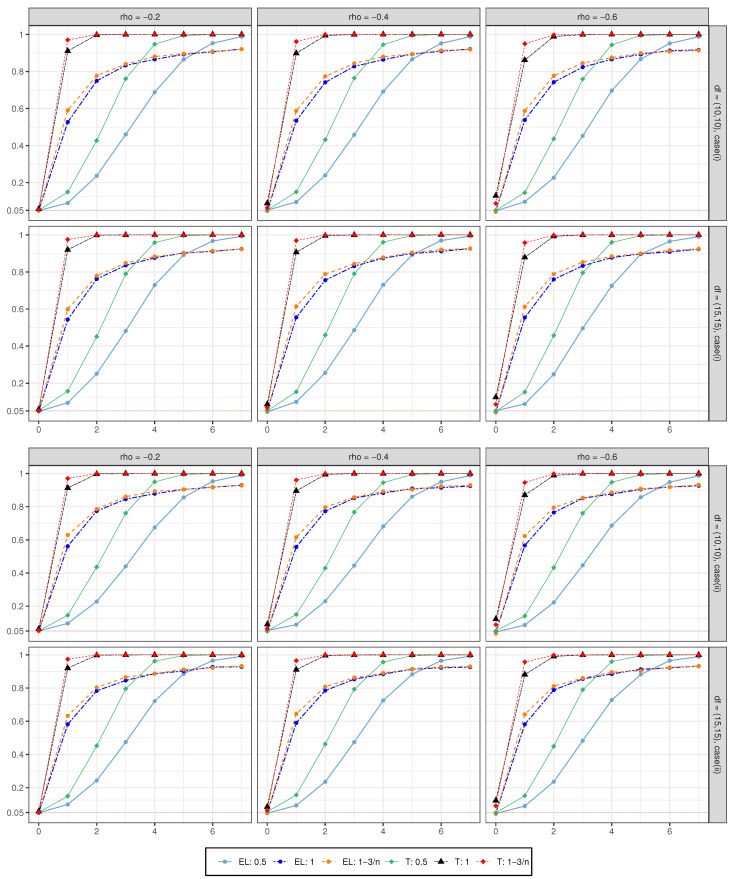
The power performance with μ=0.

**Figure 2:**
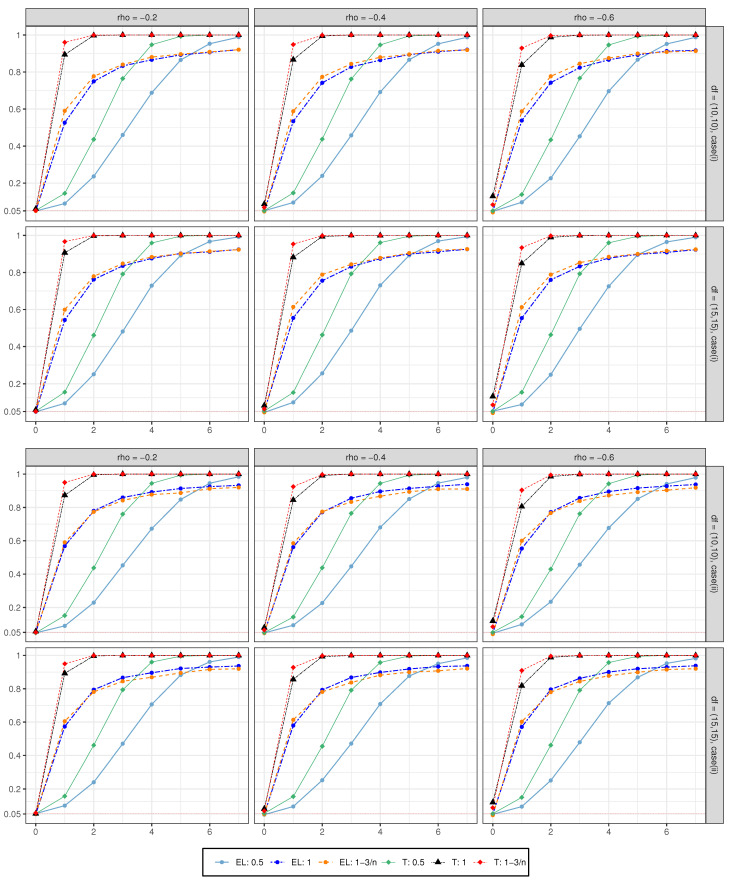
The power performance with μ=0.01.

We also generate data with $\alpha =\frac{d_{0}}{\sqrt{n}}$ (stationary) or $\alpha =\frac{d_{0}log\left(n\right)}{\sqrt{n}}$ (unit root or near the unit root), $d_{0}=\left\{0,1,2,\cdots ,7\right\}$, and other parameters are set the same as **Case (i)** and **Case (ii)**, in order to investigate the power performance of the proposed method. The results are shown in Figure 1 (μ=0) and Figure 2 (μ=0.01). Here, we only show the case where n=1000. The abscissa represents the constant $d_{0}$ value, and the ordinate is the power value. Different colors represent different processes of $X_{t}$ and tests. We can see that, under all settings, Figure 1 and Figure 2 show the same performance. Firstly, as the value of $d_{0}$ increases, the power converges quickly to 1. Secondly, when $X_{t}$ is a near unit root, the convergence rate is better than the unit root, and the unit root is better than the stationary. Last but not least, when $d_{0}=0$, it can be seen that the value of the *t*-test is significantly higher than the significance level of 0.05 under all settings. This also indicates the poor size performance of the *t*-test, even though it has a good convergence rate in power performance.

In summary, the proposed method has good size and power performance when checking whether intercept α exists and also has good robustness for different innovative distributions. The traditional *t*-test shows over-reject, which makes it easy to misjudge in real data analysis; therefore, the profile empirical likelihood method proposed in this paper is more recommended when real data are endogenous.

## 4. A Real Data Application

In this section, we illustrate our testing method on U.S. equity data, which has been explored by numerous scholars, such as [10,12,24], etc. We collected the data coming from the Center for Research in Security Prices (CRSP), and the sample period is January 1952 to December 2015 with monthly data (n=1068). Among them, the value-weighted excess returns of the S&P 500 index are used as the predicted variable $Y_{t}$, and the other 10 financial variables are dividend–price ratio, dividend yield, earnings–price ratio, dividend payout ratio, book-to-market value ratio, T-bill rate, default yield spread, long-term yield, term spread, and net equity expansion, which are used as predicting variables, respectively.

We know that predictability is one of the critical concerns in various fields, including finance, climatology, tourism, sociology, and so on (see, e.g., [25,26,27]). However, as [10] shows, a small intercept can change the predictability dramatically when the predicting variable is nearly integrated. Hence, the intercept term test is an important pre-test before the statistical inference. On the other hand, Refs. [12,28] showed that the innovation of financial data generally contains endogeneity, which will bring bias to relevant estimates. Therefore, it is undoubtedly very vital to obtain a unified test for the intercept test, no matter whether the predictive variables are stationary, nearly integrated, unit root, or innovation have endogeneity.

Firstly, we use the adf.test function in the R package to test the stationarity of each predictor variable. Then, the least square estimation was used to fit the model (2), in which $V_{t}$ is estimated based on the $V_{t}=e_{t}-\sum_{i=1}^{12}b_{i}e_{t-i}$, in order to make $V_{t}$ uncorrelated. In addition, the correlation coefficient between $U_{t}$ and $V_{t}$ was tested. Before performing our test, the ArchTest function was also used to test whether $V_{t}$ has a GARCH structure. Finally, the method proposed in this paper is used to test $H_{0}:\alpha =0$. Table 3 reports all the results for each predictor variable. The first column is the predictive variables. The second column is the estimators of intercept terms, and the third column is the estimators of the autoregressive coefficient of predictive variables. The fourth column is the *p*-values of the ADF test. The fifth column is the correlation coefficient between the two innovations $U_{t}$ and $V_{t}$. The sixth column is the *p*-values of the GARCH test of $V_{t}$ sequence. The last two columns are the *p*-values of the test proposed in this paper and the traditional *t*-test.

From Table 3, we can see that the $\hat{\phi }$ of all variables are very close to 1, and the adf.test shows that only T-bill rate and long-term yield do not reject the null hypothesis of unit root, indicating that these variables are non-stationary. The results of the correlation coefficient show that $U_{t}$ and $V_{t}$ have different degrees of correlation under different predictive variables, and we have also carried out corresponding settings in the simulation. In addition, the results of the ArchTest test show that $V_{t}$ under all predictor variables has a significant ARCH effect. The $\hat{\alpha }$ of ten variables obtained by the least square estimation are close to 0. The *t*-test showed that the intercept of Book-to-market, Default yield spread, and Term spread are 0. However, the results of the EL test are quite different from *t*-test, which shows that only the intercept of the Dividend payout ratio is not 0.

Overall, the *t*-test rejected the original hypothesis of alpha = 0 under most variables, which is consistent with the performance in the simulation. Notice that Ref. [12] mentioned that the *t*-test does not perform well when the predictor variable is persistent and its innovations are highly correlated. Furthermore, all the variables have a GARCH structure, which also will affect the *t*-test. In addition, Table 1 in [29] indicates the size distortion of the *t*-test for plausible parameter values. Hence, we recommend the EL test proposed in this paper over the traditional test.

## 5. Conclusions

Predictability testing is a common problem in the analysis of real data such as in the economic and financial fields. However, the validity of most existing tests depends on the assumption that the intercept term of the model exists. Therefore, the intercept term test is an important pre-test in practical application. However, it is worth noting that heteroscedasticity and endogeneity are common characteristics of financial data, which makes the existing intercept term test not perform well. In this paper, we develop a unified intercept test based on the empirical likelihood method under the balanced prediction regression model. The asymptotic distribution of the EL test is proved to be chi-squared, and the local power under stationary and non-stationary conditions is also proved. Simulation results verify the unified and effectiveness of the test and are superior to the *t*-test. Furthermore, in the empirical application, we performed an intercept term test on U.S. equity data. The results showed that the *t*-test rejected the null hypothesis in most variables, but the EL test shows the opposite. The relevant performance is consistent with the simulation. Therefore, when considering the intercept term test of financial data, the EL test is recommended.

## Figures and Tables

**Table 1:** The size performance of ${\left\{U_{t},V_{t}\right\}}_{t=1}^{n}$ under **Case (i)** and τ=0.05.

				EL					*t*-Test				
	ϕ	μ	ρ	200	400	800	1000	1200	200	400	800	1000	1200
df=(10,10)	0.5	0	−0.2	0.0477	0.0461	0.0514	0.0497	0.0521	0.0485	0.0525	0.0536	0.0509	0.0494
			−0.4	0.0430	0.0453	0.0486	0.0514	0.0453	0.0505	0.0493	0.0510	0.0516	0.0518
			−0.6	0.0417	0.0442	0.0495	0.0476	0.0425	0.0496	0.0521	0.0516	0.0506	0.0505
		0.01	−0.2	0.0476	0.0488	0.0501	0.0515	0.0503	0.0471	0.0474	0.0495	0.0525	0.0481
			−0.4	0.0448	0.0458	0.0477	0.0462	0.0466	0.0523	0.0536	0.0526	0.0514	0.0498
			−0.6	0.0426	0.0434	0.0442	0.0491	0.0481	0.0560	0.0499	0.0517	0.0506	0.0519
	1	0	−0.2	0.0526	0.0511	0.0483	0.0503	0.0475	0.0586	0.0615	0.0559	0.0575	0.0574
			−0.4	0.0501	0.0508	0.0502	0.0469	0.0457	0.0827	0.0836	0.0814	0.0881	0.0917
			−0.6	0.0478	0.0440	0.0445	0.0481	0.0484	0.1321	0.1246	0.1333	0.1289	0.1329
		0.01	−0.2	0.0558	0.0508	0.0506	0.0472	0.0519	0.0552	0.0587	0.0574	0.0607	0.0594
			−0.4	0.0483	0.0506	0.0473	0.0486	0.0498	0.0827	0.0805	0.0843	0.0867	0.0825
			−0.6	0.0488	0.0493	0.0493	0.0432	0.0443	0.1277	0.1208	0.1184	0.1292	0.1229
	1−$\frac{3}{n}$	0	−0.2	0.0484	0.0468	0.0465	0.0502	0.0512	0.0533	0.0513	0.0544	0.0550	0.0543
			−0.4	0.0475	0.0507	0.0453	0.0467	0.0440	0.0683	0.0707	0.0679	0.0634	0.0703
			−0.6	0.0463	0.0389	0.0366	0.0422	0.0403	0.0911	0.0893	0.0845	0.0876	0.0846
		0.01	−0.2	0.0507	0.0481	0.0489	0.0496	0.0487	0.0513	0.0578	0.0526	0.0522	0.0505
			−0.4	0.0467	0.0501	0.0458	0.0454	0.0470	0.0695	0.0682	0.0677	0.0690	0.0658
			−0.6	0.0386	0.0409	0.0403	0.0397	0.0415	0.0869	0.0898	0.0898	0.0835	0.0868
df=(15,15)	0.5	0	−0.2	0.0421	0.0497	0.0495	0.0486	0.0486	0.0471	0.0495	0.0495	0.0529	0.0495
			−0.4	0.0427	0.0455	0.0463	0.0455	0.0499	0.0463	0.0528	0.0490	0.0516	0.0505
			−0.6	0.0361	0.0439	0.0429	0.0519	0.0486	0.0549	0.0480	0.0531	0.0477	0.0527
		0.01	−0.2	0.0446	0.0498	0.0453	0.0497	0.0496	0.0499	0.0541	0.0523	0.0514	0.0517
			−0.4	0.0435	0.0462	0.0460	0.0455	0.0471	0.0512	0.0457	0.0499	0.0528	0.0504
			−0.6	0.0417	0.0444	0.0460	0.0451	0.0488	0.0477	0.0518	0.0513	0.0508	0.0528
	1	0	−0.2	0.0494	0.0511	0.0519	0.0497	0.0448	0.0566	0.0617	0.0567	0.0584	0.0572
			−0.4	0.0542	0.0496	0.0505	0.0527	0.0497	0.0839	0.0881	0.0914	0.0862	0.0798
			−0.6	0.0506	0.0492	0.0454	0.0464	0.0432	0.1226	0.1266	0.1282	0.1244	0.1248
		0.01	−0.2	0.0518	0.0469	0.0514	0.0533	0.0498	0.0632	0.0568	0.0607	0.0587	0.0588
			−0.4	0.0533	0.0481	0.0486	0.0486	0.0472	0.0802	0.0818	0.0829	0.0823	0.0821
			−0.6	0.0483	0.0463	0.0468	0.0482	0.0435	0.1284	0.1275	0.1224	0.1317	0.1304
	1−$\frac{3}{n}$	0	−0.2	0.0511	0.0468	0.0502	0.0511	0.0508	0.0502	0.0546	0.0534	0.0525	0.0514
			−0.4	0.0476	0.0476	0.0476	0.0457	0.0419	0.0657	0.0635	0.0670	0.0685	0.0677
			−0.6	0.0436	0.0412	0.0435	0.0422	0.0408	0.0908	0.0831	0.0890	0.0866	0.0886
		0.01	−0.2	0.0539	0.0500	0.0481	0.0472	0.0492	0.0559	0.0545	0.0561	0.0506	0.0565
			−0.4	0.0467	0.0474	0.0427	0.0476	0.0428	0.0678	0.0681	0.0659	0.0641	0.0702
			−0.6	0.0428	0.0445	0.0438	0.0410	0.0417	0.0855	0.0844	0.0864	0.0857	0.0840

**Table 2:** The size performance of ${\left\{U_{t},V_{t}\right\}}_{t=1}^{n}$ under **Case (ii)** and τ=0.05.

				EL					*t*-Test				
	ϕ	μ	ρ	200	400	800	1000	1200	200	400	800	1000	1200
df=(10,10)	0.5	0	−0.2	0.0484	0.0470	0.0510	0.0503	0.0547	0.0505	0.0509	0.0507	0.0515	0.0509
			−0.4	0.0402	0.0420	0.0500	0.0531	0.0500	0.0490	0.0523	0.0479	0.0499	0.0502
			−0.6	0.0406	0.0382	0.0465	0.0432	0.0470	0.0532	0.0520	0.0532	0.0507	0.0500
		0.01	−0.2	0.0434	0.0435	0.0481	0.0480	0.0497	0.0527	0.0475	0.0493	0.0476	0.0508
			−0.4	0.0422	0.0462	0.0485	0.0472	0.0439	0.0506	0.0463	0.0519	0.0486	0.0492
			−0.6	0.0418	0.0419	0.0452	0.0454	0.0464	0.0524	0.0481	0.0483	0.0507	0.0500
	1	0	−0.2	0.0514	0.0484	0.0534	0.0511	0.0499	0.0565	0.0601	0.0606	0.0618	0.0566
			−0.4	0.0533	0.0473	0.0520	0.0515	0.0511	0.0834	0.0872	0.0858	0.0896	0.0864
			−0.6	0.0497	0.0497	0.0470	0.0452	0.0503	0.1263	0.1228	0.1279	0.1218	0.1265
		0.01	−0.2	0.0536	0.0506	0.0493	0.0513	0.0461	0.0581	0.0597	0.0556	0.0558	0.0577
			−0.4	0.0523	0.0476	0.0515	0.0479	0.0501	0.0802	0.0817	0.0814	0.0788	0.0770
			−0.6	0.0481	0.0448	0.0465	0.0467	0.0464	0.1193	0.1234	0.1198	0.1187	0.1133
	1−$\frac{3}{n}$	0	−0.2	0.0533	0.0492	0.0489	0.0488	0.0492	0.0502	0.0540	0.0536	0.0552	0.0554
			−0.4	0.0522	0.0439	0.0469	0.0487	0.0472	0.0675	0.0704	0.0691	0.0641	0.0659
			−0.6	0.0445	0.0442	0.0445	0.0348	0.0406	0.0881	0.0869	0.0847	0.0886	0.0885
		0.01	−0.2	0.0513	0.0493	0.0504	0.0489	0.0472	0.0574	0.0512	0.0550	0.0505	0.0580
			−0.4	0.0477	0.0467	0.0488	0.0461	0.0454	0.0654	0.0635	0.0615	0.0650	0.0658
			−0.6	0.0464	0.0429	0.0404	0.0401	0.0422	0.0870	0.0868	0.0853	0.0845	0.0873
df=(15,15)	0.5	0	−0.2	0.0457	0.0471	0.0498	0.0487	0.0453	0.0491	0.0503	0.0536	0.0513	0.0523
			−0.4	0.0419	0.0436	0.0435	0.0462	0.0463	0.0499	0.0519	0.0482	0.0545	0.0490
			−0.6	0.0393	0.0430	0.0444	0.0439	0.0464	0.0542	0.0527	0.0516	0.0488	0.0559
		0.01	−0.2	0.0438	0.0479	0.0457	0.0525	0.0484	0.0515	0.0468	0.0536	0.0523	0.0485
			−0.4	0.0394	0.0431	0.0479	0.0459	0.0483	0.0486	0.0486	0.0524	0.0536	0.0516
			−0.6	0.0361	0.0426	0.0443	0.0481	0.0462	0.0499	0.0514	0.0487	0.0520	0.0478
	1	0	−0.2	0.0507	0.0546	0.0518	0.0482	0.0492	0.0563	0.0600	0.0554	0.0572	0.0583
			−0.4	0.0560	0.0490	0.0501	0.0503	0.0501	0.0803	0.0841	0.0840	0.0847	0.0842
			−0.6	0.0521	0.0508	0.0445	0.0461	0.0455	0.1322	0.1318	0.1294	0.1225	0.1258
		0.01	−0.2	0.0529	0.0489	0.0506	0.0490	0.0514	0.0570	0.0584	0.0565	0.0522	0.0587
			−0.4	0.0510	0.0469	0.0494	0.0487	0.0494	0.0780	0.0812	0.0793	0.0808	0.0815
			−0.6	0.0454	0.0491	0.0501	0.0486	0.0454	0.1237	0.1223	0.1135	0.1195	0.1121
	1−$\frac{3}{n}$	0	−0.2	0.0513	0.0478	0.0490	0.0505	0.0522	0.0541	0.0515	0.0537	0.0534	0.0523
			−0.4	0.0485	0.0484	0.0458	0.0461	0.0471	0.0675	0.0674	0.0629	0.0651	0.0664
			−0.6	0.0447	0.0425	0.0410	0.0421	0.0413	0.0860	0.0851	0.0896	0.0903	0.0860
		0.01	−0.2	0.0527	0.0540	0.0485	0.0495	0.0486	0.0571	0.0566	0.0554	0.0563	0.0539
			−0.4	0.0545	0.0501	0.0445	0.0482	0.0450	0.0623	0.0690	0.0653	0.0684	0.0639
			−0.6	0.0491	0.0465	0.0407	0.0429	0.0478	0.0874	0.0820	0.0833	0.0871	0.0849

**Table 3:** Test results of 10 financial variables.

Predictor	$\hat{\alpha }$	$\hat{\phi }$	*Adf.Test*	Cor($U_{t}$, $V_{t}$)	GARCH-V	EL	*t*-Test
Dividend–price ratio	0.0319	0.9928	0.0157 **	−0.9618	$9.6402\times 10^{-37}$ ***	0.4200	0.0102 **
Dividend yield	0.0357	0.9929	0.0239 **	−0.0791	$5.4329\times 10^{-34}$ ***	0.4626	0.0042 ***
Earnings–price ratio	0.0308	0.9870	0.0112 **	−0.7966	$7.6498\times 10^{-35}$ ***	0.2730	0.0058 ***
Dividend payout ratio	0.0072	0.9913	0.0100 ***	−0.0407	$1.7194\times 10^{-41}$ ***	$0.0828^{*}$	0.0487 **
Book-to-market value ratio	−0.0050	0.9858	0.0100 ***	−0.8031	$1.1404\times 10^{-84}$ ***	0.1322	0.2146
T-bill rate	0.0097	0.9934	0.5266	-0.0762	$8.1443\times 10^{-68}$ ***	0.2945	0.0001 ***
Default yield spread	0.0013	0.9752	0.0100 ***	−0.2433	$3.4015\times 10^{-72}$ ***	0.2027	0.6769
Long-term yield	0.0105	0.9965	0.9568	−0.1098	$2.0058\times 10^{-44}$ ***	0.2722	0.0033 **
Term spread	0.0033	0.9608	0.0100 ***	−0.0070	$8.4860\times 10^{-43}$ ***	0.2126	0.2350
Net equity expansion	0.0090	0.9805	0.0100 ***	−0.0619	$3.1793\times 10^{-39}$ ***	0.7741	$1.43\times 10^{-5}$ ***

Significance levels: * *p* ≤ 0.1, ** *p* ≤ 0.05, *** *p* ≤ 0.01.

## Data Availability

The data used in applications can be found in the Center for Research in Security Prices.

## Appendix A. Detailed Proofs of the Main Results

In this appendix, we provide detailed proofs for the main results presented in Section 2. Before proceeding further, we specify some useful lemmas as follows:

**Lemma** **A1.**
*Under the same conditions of Theorem 1, we have as n→∞ that:*

(a) *maxp+1≤t≤msupβψ∈B∥Zt(α0,β,ψ)∥=op(m),*
(b) *$\frac{1}{\sqrt{m}}\sum_{t=p+1}^{m}Z_{t}\left(\alpha_{0},\beta ,\psi \right)=\frac{1}{\sqrt{m}}\sum_{t=p+1}^{m}Z_{t}\left(\alpha_{0},\beta_{0},\psi_{0}\right)+O_{p}\left(1\right)$ holds uniformly for βψ∈B,*
(c) *$\frac{1}{m}\sum_{t=p+1}^{m}\left\{Z_{t}\left(\alpha_{0},\beta ,\psi \right)Z_{t}^{⊤}\left(\alpha_{0},\beta ,\psi \right)\right\}=\frac{1}{m}\sum_{t=p+1}^{m}\left\{Z_{t}\left(\alpha_{0},\beta_{0},\psi_{0}\right)Z_{t}^{⊤}\left(\alpha_{0},\beta_{0},\psi_{0}\right)\right\}+o_{p}\left(1\right)$ holds uniformly for βψ∈B,*

*where B={βψ:m(k−1)/2|β−β0|+|ψ−ψ0|≤Cm} for some constant C>0, and k=1,2,3 correspond to* ***Cases (i)–(iii)*** *, respectively.*

**Proof of Lemma** **A1.** In the sequel, we only prove **Case (ii)**, as the rest of the cases can be proved similarly. We first prove Part (a). Note that, by [30], we have for any s∈(0,2] that

(A1) $$\begin{array}{c} \frac{1}{\sqrt{m}}X_{\left[ms\right]}\overset{D}{\Rightarrow }J_{c}\left(s\right):=\int_{0}^{s}e^{-(s-r)c}dW\left(r\right), \end{array}$$

for **Case (ii)** as n→∞, where ‘$\overset{D}{\Rightarrow }$’ stands for the convergence in space, and D(0,1] is the space of real-valued functions on the interval (0,1] that are right continuous and have finite left limits. Since

maxp+1≤t≤msupβψ∈B∥Zt(α0,β,ψ)∥≤∑k=1p+2maxp+1≤t≤msupβψ∈B|Zt,k(α0,β,ψ)|,

we need only to check the upper bound for each component $Z_{t,k}\left(\alpha_{0},\beta ,\psi \right)$. For the first component, rewrite $Z_{t,1}\left(\alpha_{0},\beta ,\psi \right)=\varepsilon_{t}-\sqrt{m}\left(\beta -\beta_{0}\right)\frac{1}{\sqrt{m}}X_{t-1}-\sum_{i=1}^{p}\left(\psi_{i}-\psi_{i0}\right)\Delta X_{t-i}$. Trivially, based on Conditions C1–C2, we have $\max_{p+1\leq t\leq 2m}\left|\varepsilon_{t}\right|=o_{p}\left(\sqrt{m}\right)$. Next, using (A1), it is easy to check for any ϵ>0 that

$$\begin{array}{ccc} P\left(\max_{p+1\leq t\leq 2m}\left|\frac{1}{\sqrt{m}}X_{t-1}\right|\geq \epsilon \sqrt{m}\right) & \leq & \frac{2}{\epsilon^{2+\delta }m^{\delta }}\frac{1}{2m}E\left(\sum_{t=1}^{2m}{\left|\frac{1}{\sqrt{m}}X_{t-1}\right|}^{2+\delta }\right)\\ & \to & \frac{2}{\epsilon^{2+\delta }m^{\delta }}E\left(\int_{0}^{2}{\left|J_{c}\left(s\right)\right|}^{2+\delta }ds\right)\to 0, \end{array}$$

which implies $\max_{1\leq t\leq 2m}\left|\frac{1}{\sqrt{m}}X_{t-1}\right|=o_{p}\left(\sqrt{m}\right)$, and in turn $\max_{1\leq t\leq 2m}\left|\left(\beta -\beta_{0}\right)X_{t-1}\right|=o_{p}\left(1\right)$ uniformly for βψ∈B. Using this, by $\Delta X_{t}=X_{t}-X_{t-1}=\frac{c}{n}X_{t-1}+\varepsilon_{t}$, we have as n→∞ that

$$\begin{array}{c} \max_{1\leq t\leq 2m}|\Delta X_{t}|\leq \frac{c\sqrt{m}}{n}\max_{1\leq t\leq 2m}|\frac{1}{\sqrt{m}}X_{t}|+\max_{1\leq t\leq 2m}|\varepsilon_{t}|=\max_{1\leq t\leq 2m}\left|\varepsilon_{t}\right|\cdot \left\{1+o_{p}\left(1\right)\right\}, \end{array}$$

and hence

$$\begin{array}{c} \max_{p+1\leq t\leq 2m}\left|\sum_{i=1}^{p}\left(\psi_{i}-\psi_{i0}\right)\Delta X_{t-i}\right|=o_{p}\left(1\right), \end{array}$$

uniformly for βψ∈B. This shows

$$\begin{array}{c} \max_{p+1\leq t\leq m}\underset{B}{\sup}|Z_{t,1}\left(\alpha_{0},\beta ,\psi \right)|\leq \max_{1\leq t\leq m}\left|\varepsilon_{t}\right|\cdot \left\{1+o_{p}\left(1\right)\right\}. \end{array}$$

Similarly, we can show that $\max_{p+1\leq t\leq m}\sup_{B}|Z_{t,l}\left(\alpha_{0},\beta ,\psi \right)|\leq \max_{1\leq t\leq m}\left|\varepsilon_{t}\right|\cdot \left\{1+o_{p}\left(1\right)\right\}$, for l=2,⋯,p+2. This shows Part (a). For Part (b), note that

(A2) $$\begin{array}{c} Z_{t}\left(\alpha_{0},\beta ,\psi \right)=Z_{t}\left(\alpha_{0},\beta_{0},\psi_{0}\right)-\left(\beta -\beta_{0}\right){\tilde{X}}_{t-1}-\sum_{i=1}^{p}\left(\psi_{i}-\psi_{i,0}\right){\tilde{X}}_{t-i}^{*}, \end{array}$$

where ${\tilde{X}}_{t-1}={\left(X_{t-1},X_{t+m-1}w_{t+m},X_{t-1},\cdots ,X_{t-1}\right)}^{⊤}$, and ${\tilde{X}}_{t-i}^{*}=(\Delta X_{t-i},\Delta X_{t+m-i}w_{t+m}$, $\Delta X_{t-i}\Delta X_{t-1},\cdots ,\Delta X_{t-i}\Delta X_{t-p}{)}^{⊤}$. Then, by using a similar proof to that of Part (a), we have

$$\begin{array}{cc} & m\left(\beta -\beta_{0}\right)\cdot \frac{1}{m}\sum_{t=1}^{m}\frac{1}{\sqrt{m}}{\tilde{X}}_{t-1}=O_{p}\left(1\right),\;\mathrm{and}\;\\ & \sum_{i=1}^{p}\left\{\sqrt{m}\left(\psi_{i}-\psi_{i,0}\right)\cdot \frac{1}{m}\sum_{t=1}^{m}{\tilde{X}}_{t-i}^{*}\right\}=O_{p}\left(1\right), \end{array}$$

uniformly for βψ∈B as n→∞. The proof of Part (c) is similar to that of Part (b) based on (A2). We omit the details. This completes the proof of Lemma A1. □

**Lemma** **A2.** *Under the same conditions of Theorem 1, we have as n→∞ that:*

(a) *$\frac{1}{\sqrt{m}}\sum_{t=p+1}^{m}Z_{t}\left(\alpha_{0},\beta_{0},\psi_{0}\right)\overset{d}{⟶}N\left(0,\Sigma \right)$, and*
(b) *$\frac{1}{m}\sum_{t=p+1}^{m}\left\{Z_{t}\left(\alpha_{0},\beta_{0},\psi_{0}\right)Z_{t}^{⊤}\left(\alpha_{0},\beta_{0},\psi_{0}\right)\right\}\overset{p}{⟶}\Sigma$,*

*where ‘$\overset{p}{⟶}$’ denotes the convergence in probability. For****Case (i)****,* Σ *is specified in Theorem 2, and* ***Cases (ii)*** *and* ***(iii)*** *,*

$$\begin{array}{c} \Sigma =\left(\begin{array}{ccc} E(U_{1}^{2}) & & \\ & {\bar{\sigma }}^{2} & \\ & & \tilde{\Gamma } \end{array}\right)\;\;\;with\;\tilde{\Gamma }=E\left(U_{1}^{2}\right)\left(\begin{array}{ccc} \mu^{2}+\lim_{t\to \infty }E\left(e_{t-1}^{2}\right) & & \\ & \ldots & \\ & & \mu^{2}+\lim_{t\to \infty }E\left(e_{t-p}^{2}\right) \end{array}\right). \end{array}$$

*Note that μ=0 for **Case (ii)***.

**Proof of Lemma** **A2.** We only prove Part (a), as Part (b) follows a similar fashion. Put

$$\begin{array}{c} Z_{t}\left(\alpha_{0},\beta_{0},\psi_{0}\right)={\left(U_{t},U_{t}w_{t}+{\bar{\delta }}_{t},U_{t}\Delta X_{t-1},\cdots ,U_{t}\Delta X_{t-p}\right)}^{⊤}. \end{array}$$

Denote $F_{t}$ as the sigma field generated by $\left\{\left(U_{s},e_{s}\right):1\leq s\leq t,m+1\leq s\leq t+m\right\}$. Then, it is easy to check that $\left\{Z_{t}\left(\alpha_{0},\beta_{0},\psi_{0}\right)\right\}$ is a Martingale difference array with respect to the filtration $F_{t}$. For **Case (i)**, noting that $\left\{X_{t}\right\}$ is stationary, the normality of $\frac{1}{\sqrt{m}}\sum_{t=p+1}^{m}Z_{t}\left(\alpha_{0},\beta_{0},\psi_{0}\right)$ follows immediately under Conditions C1–C3 by the center limit theory for Martingale differences of [31] and the Cramér–Wold device. For **Case (ii)**, trivially, we have

$$\frac{1}{\sqrt{m}}\sum_{t=p+1}^{m}Z_{t,1}\left(\alpha_{0},\beta_{0},\psi_{0}\right)\overset{d}{⟶}N\left(0,E\left(U_{1}^{2}\right)\right),\;\;\mathrm{as}\;n\to \infty .$$

Next, note that $|X_{t}|\overset{p}{⟶}\infty$, which results in $\frac{1}{m}\sum_{t=1}^{m}w_{t}^{2}\overset{p}{⟶}0$ as n→∞. Hence,

$$\begin{array}{ccc} \frac{1}{\sqrt{m}}\sum_{t=p+1}^{m}Z_{t,2}\left(\alpha_{0},\beta_{0},\psi_{0}\right) & = & \frac{1}{\sqrt{m}}\sum_{t=p+1}^{m}U_{t+1}w_{t+m-1}+\frac{1}{\sqrt{m}}\sum_{t=p+1}^{m}{\bar{\delta }}_{t}\\ & = & \frac{1}{\sqrt{m}}\sum_{t=p+1}^{m}{\bar{\delta }}_{t}+o_{p}\left(1\right), \end{array}$$

which implies $\frac{1}{\sqrt{m}}\sum_{t=p+1}^{m}Z_{t,2}\left(\alpha_{0},\beta_{0},\psi_{0}\right)\overset{d}{⟶}N\left(0,{\bar{\sigma }}^{2}\right)$. For $\frac{1}{\sqrt{m}}\sum_{t=p+1}^{m}Z_{t,2+i}\left(\alpha_{0},\beta_{0},\psi_{0}\right),k=1,2,\cdots ,p$, by using a similar proof to [30], we have

(A3) $$\begin{array}{ccc} & & \frac{1}{m}\sum_{t=p+1}^{m}E\left(Z_{t,2+i}^{2}\left(\alpha_{0},\beta_{0},\psi_{0}\right)|F_{t-1}\right)\\ & = & E\left(U_{1}^{2}\right)\frac{1}{m}\sum_{t=p+1}^{m}{\left(\Delta X_{t-i}\right)}^{2}\\ & = & E\left(U_{1}^{2}\right)\frac{1}{m}\sum_{t=p+1}^{m}{\left(\frac{c}{n}X_{t-i-1}+e_{t-i}\right)}^{2}\\ & = & E\left(U_{1}^{2}\right)\left\{\frac{c^{2}}{n}\frac{1}{m}\sum_{t=p+1}^{m}{\left(\frac{1}{\sqrt{n}}X_{t-i-1}\right)}^{2}+\frac{2c}{\sqrt{n}}\frac{1}{m}\sum_{t=p+1}^{m}\frac{X_{t-i-1}e_{t-i}}{\sqrt{n}}+\frac{1}{m}\sum_{t=p+1}^{m}e_{t-i}^{2}\right\}\\ & = & E\left(U_{1}^{2}\right)\left\{\frac{c^{2}}{n}\int_{0}^{1}J_{c}^{2}\left(s\right)ds+\frac{2c}{\sqrt{n}}\frac{1}{\sqrt{m}}\int_{0}^{1}J_{c}\left(s\right)dW\left(s\right)+\lim_{t\to \infty }E\left(\varepsilon_{t-i}^{2}\right)\right\}\\ & = & E\left(U_{1}^{2}\right)\cdot \lim_{t\to \infty }E\left(\varepsilon_{t-i}^{2}\right)\cdot \left\{1+o_{p}\left(1\right)\right\},\;\mathrm{as}\;n\to \infty . \end{array}$$

and, for arbitrarily small $d_{1},d_{2}\in \left(0,\epsilon_{0}\right)$,

$$\begin{array}{ccc} & & \frac{1}{m}\sum_{t=p+1}^{m}E(|Z_{t,2+i}\left(\alpha_{0},\beta_{0},\psi_{0}\right){|}^{2}I(|Z_{t,2+i}\left(\alpha_{0},\beta_{0},\psi_{0}\right)|\geq d_{1}\sqrt{m})|F_{t-1})\\ & \leq & \frac{1}{d_{1}^{d_{2}}m^{d_{2}/2}}\frac{1}{m}\sum_{t=p+1}^{m}E(|Z_{t,2+i}\left(\alpha_{0},\beta_{0},\psi_{0}\right){|}^{2+d_{2}}\left|F_{t-1}\right)\\ & = & \frac{E(|U_{1}{|}^{2+d_{2}})}{d_{1}^{d_{2}}m^{d_{2}/2}}\frac{1}{m}\sum_{t=p+1}^{m}{\left|\frac{c}{n}X_{t-i-1}+e_{t-i}\right|}^{2+d_{2}}\\ & \leq & \frac{E(|U_{1}{|}^{2+d_{2}})2^{1+d_{2}}}{d_{1}^{d_{2}}m^{d_{2}/2}}\left\{\frac{1}{m}\sum_{t=p+1}^{m}{\left|\frac{c}{n}X_{t-i-1}\right|}^{2+d_{2}}+\frac{1}{m}\sum_{t=p+1}^{m}{\left|e_{t-i}\right|}^{2+d_{2}}\right\}\\ & = & \frac{E(|U_{1}{|}^{2+d_{2}})2^{1+d_{2}}}{d_{1}^{d_{2}}m^{d_{2}/2}}\frac{1}{m}\sum_{t=p+1}^{m}{\left|e_{t-i}\right|}^{2+d_{2}}\cdot \left\{1+o_{p}\left(1\right)\right\}\overset{p}{⟶}0,\;\;\mathrm{as}\;n\to \infty . \end{array}$$

Based on this and (A3), a simple application of the center limit theory for Martingale differences of [31] leads to the fact that, as n→∞,

$$\begin{array}{c} \frac{1}{\sqrt{m}}\sum_{t=p+1}^{m}Z_{t,2+i}\left(\alpha_{0},\beta_{0},\psi_{0}\right)\overset{d}{⟶}N\left(0,E\left(U_{1}^{2}\right)\cdot \lim_{t\to \infty }E\left(\varepsilon_{t-i}^{2}\right)\right),\;\;\;k=1,2,\cdots ,p. \end{array}$$

By noting that $\left\{{\bar{\delta }}_{t}\right\}$ is independent of the random observations, we can show that

$$\begin{array}{c} \frac{1}{m}\sum_{t=p+1}^{m}E\left(Z_{t,2}\left(\alpha_{0},\beta_{0},\psi_{0}\right)Z_{t,j}\left(\alpha_{0},\beta_{0},\psi_{0}\right)|F_{t-1}\right)\overset{p}{⟶}0, \end{array}$$

for j≠2. Similar to (A3), it is easy to check that

$$\begin{array}{c} \frac{1}{m}\sum_{t=p+1}^{m}E\left(Z_{t,i}\left(\alpha_{0},\beta_{0},\psi_{0}\right)Z_{t,j}\left(\alpha_{0},\beta_{0},\psi_{0}\right)|F_{t-1}\right)\overset{p}{⟶}0, \end{array}$$

for i,j≠2 as n→∞. Hence, Part (a) follows immediately by using the Cramér–Wold device. This completes the proof of this lemma. □

**Proof of Theorem** **1.** We only prove **Case (ii)** as **Cases (i)** and **(iii)** are similar. For convenience, denote

$$\begin{array}{ccc} h(\alpha_{0},\beta ,\psi ,x) & = & \frac{1}{m}\sum_{t=p+1}^{m}\frac{Z_{t}\left(\alpha_{0},\beta ,\psi \right)}{1+x^{⊤}Z_{t}\left(\alpha_{0},\beta ,\psi \right)},\;\mathrm{and}\;\\ g_{t}\left(\alpha_{0},\beta ,\psi \right) & = & \lambda^{⊤}Z_{t}\left(\alpha_{0},\beta ,\psi \right) \end{array}$$

with λ being the solution to $h(\alpha_{0},\beta ,\psi ,x)=0$ with respect to x for fixed β,ψ. The following proof is similar to that of Theorem 1 in [32]. Let λ=ϱv with ϱ=∥λ∥. Then, similar to [33], we have $p_{t}=\frac{1}{m}\frac{1}{1+\lambda^{⊤}Z_{t}\left(\alpha_{0},\beta ,\psi \right)}$, t=p+1,⋯,m, by using the Lagrange multipliers method. Note that

$$\begin{array}{ccc} 0 & = & |\lambda^{⊤}h\left(\alpha_{0},\beta ,\psi ,\lambda \right)|\\ & = & \left|\frac{1}{m}\sum_{t=p+1}^{m}g_{t}\left(\alpha_{0},\beta ,\psi \right)-\frac{1}{m}\sum_{t=p+1}^{m}\frac{g_{t}^{2}\left(\alpha_{0},\beta ,\psi \right)}{1+g_{t}\left(\alpha_{0},\beta ,\psi \right)}\right|\\ & \geq & \left|\frac{1}{m}\sum_{t=p+1}^{m}\frac{g_{t}^{2}\left(\alpha_{0},\beta ,\psi \right)}{1+g_{t}\left(\alpha_{0},\beta ,\psi \right)}\right|-\left|\frac{1}{m}\sum_{t=p+1}^{m}g_{t}\left(\alpha_{0},\beta ,\psi \right)\right|\\ & \geq & \frac{1}{m}\sum_{t=p+1}^{m}\frac{g_{t}^{2}\left(\alpha_{0},\beta ,\psi \right)}{1+\max_{p+1\leq t\leq m}g_{t}\left(\alpha_{0},\beta ,\psi \right)}-\left|\frac{1}{m}\sum_{t=p+1}^{m}g_{t}\left(\alpha_{0},\beta ,\psi \right)\right|. \end{array}$$

Then, by Lemmas 1 and 2, we have

$$\begin{array}{ccc} & & {∥\lambda ∥}^{2}\frac{v^{⊤}\frac{1}{m}\sum_{t=p+1}^{m}Z_{t}\left(\alpha_{0},\beta ,\psi \right)Z_{t}{\left(\alpha_{0},\beta ,\psi \right)}^{⊤}v}{1+\max_{p+1\leq t\leq m}g_{t}\left(\alpha_{0},\beta ,\psi \right)}\\ & \leq & \left|\frac{1}{m}\sum_{t=p+1}^{m}g_{t}\left(\alpha_{0},\beta ,\psi \right)\right|\\ & \leq & \left|\lambda^{⊤}\frac{1}{m}\sum_{t=p+1}^{n}Z_{t}\left(\alpha_{0},\beta_{0},\psi_{0}\right)\right|+\left|\lambda^{⊤}\frac{1}{m}\sum_{t=p+1}^{m}\left(Z_{t}\left(\alpha_{0},\beta ,\psi \right)-Z_{t}\left(\alpha_{0},\beta_{0},\psi_{0}\right)\right)\right|\\ & = & ∥\lambda ∥\times O_{p}\left(1/\sqrt{n}\right), \end{array}$$

uniformly for βψ∈B. Hence,

$$\begin{array}{c} ∥\lambda ∥=O_{p}\left(1/\sqrt{m}\right), \end{array}$$

uniformly for βψ∈B by noting that Σ is positive definite. This, together with Part (a) of Lemma A1, shows

$$\begin{array}{c} \max_{p+1\leq t\leq m}|g_{t}\left(\alpha_{0},\beta ,\psi \right)|\leq ∥\lambda ∥\times \max_{p+1\leq t\leq m}∥Z_{t}\left(\alpha_{0},\beta ,\psi \right)∥=O_{p}\left(1/\sqrt{m}\right)o_{p}\left(\sqrt{m}\right)=o_{p}\left(1\right), \end{array}$$

uniformly for βψ∈B. In turn,

$$\begin{array}{c} ∥\frac{1}{m}\sum_{t=p+1}^{m}Z_{t}\left(\alpha_{0},\beta ,\psi \right)\frac{g_{t}^{2}\left(\alpha_{0},\beta ,\psi \right)}{1+g_{t}\left(\alpha_{0},\beta ,\psi \right)}∥\leq ∥\lambda ∥\cdot a_{\max}\cdot 2\max_{p+1\leq t\leq m}g_{t}\left(\alpha_{0},\beta ,\psi \right)=o_{p}\left(1/\sqrt{m}\right), \end{array}$$

where $a_{\max}$ denotes the maximum eigenvalue of Σ. Next, by Lemma 1, we have

$$\begin{array}{ccc} 0 & = & h(\alpha_{0},\beta ,\psi ,\lambda )\\ & = & \frac{1}{m}\sum_{t=p+1}^{m}Z_{t}\left(\alpha_{0},\beta ,\psi \right)\frac{1-g_{t}^{2}\left(\alpha_{0},\beta ,\psi \right)+g_{t}^{2}\left(\alpha_{0},\beta ,\psi \right)}{1+g_{t}\left(\alpha_{0},\beta ,\psi \right)}\\ & = & \frac{1}{m}\sum_{t=p+1}^{m}Z_{t}\left(\alpha_{0},\beta ,\psi \right)\left(1-g_{t}\left(\alpha_{0},\beta ,\psi \right)\right)+\frac{1}{m}\sum_{t=p+1}^{m}Z_{t}\left(\alpha_{0},\beta ,\psi \right)\frac{g_{t}^{2}\left(\alpha_{0},\beta ,\psi \right)}{1+g_{t}\left(\alpha_{0},\beta ,\psi \right)}\\ & = & \frac{1}{m}\sum_{t=p+1}^{m}Z_{t}\left(\alpha_{0},\beta ,\psi \right)\left(1-Z_{t}{\left(\alpha_{0},\beta ,\psi \right)}^{⊤}\lambda \right)+o_{p}\left(1/\sqrt{m}\right)\\ & = & \frac{1}{m}\sum_{t=p+1}^{m}Z_{t}\left(\alpha_{0},\beta ,\psi \right)-\Sigma \lambda +o_{p}\left(1/\sqrt{m}\right), \end{array}$$

uniformly for βψ∈B. Then, we have

(A4) $$\begin{array}{c} \lambda =\Sigma^{-1}\frac{1}{m}\sum_{t=p+1}^{m}Z_{t}\left(\alpha_{0},\beta ,\psi \right)+o_{p}\left(1/\sqrt{m}\right) \end{array}$$

uniformly for βψ∈B as n→∞. As did in the proof of Theorem 1 in [32], we can show by the Taylor expansion that

(A5) $$\begin{array}{c} \underset{B}{\sup}∥-2\log L\left(\alpha_{0},\beta ,\psi \right)-\xi_{n}^{⊤}\left(\beta ,\psi \right)\Sigma^{-1}\xi_{n}\left(\beta ,\psi \right)∥=o_{p}\left(1\right),\;\;\mathrm{as}\;n\to \infty , \end{array}$$

where $\xi_{n}\left(\beta ,\psi \right)=\frac{1}{\sqrt{m}}\sum_{t=p+1}^{m}Z_{t}\left(\alpha_{0},\beta ,\psi \right)$, based on (A4) and Lemmas 1 and 2. Using this, we have that the minimizer ${\left(\hat{\beta },{\hat{\psi }}^{⊤}\right)}^{⊤}$ of $-2\log L\left(\alpha_{0},\beta ,\psi \right)+2\log L\left(\alpha_{0},\beta_{0},\psi_{0}\right)$ must lie in B. Next, for any βψ∈B, write m(β−β0)ψ−ψ0=ym with y=y1y2 and ∥y∥<C for some constant *C* given in B. Then, by (A2), a similar proof to that of Part (a) in Lemma (A1) leads to the fact that

1m∑t=p+1m(Zt(α0,β,ψ)−Zt(α0,β0,ψ0))=−1m∑t=p+1m1mX˜t−1,X˜t−1*,⋯,X˜t−p*m(β−β0)m(ψ−ψ0)+op(1)=−limt→∞∫01Jc(s)ds00⋯0000⋯00E(et−12)0⋯000E(et−22)⋯0⋮⋮⋮⋮⋮00⋯0E(et−p2)·m(β−β0)m(ψ−ψ0)+op(1):=Ay+op(1),

where ${\tilde{X}}_{t-1}$, ${\tilde{X}}_{t-i}^{*},i=1,2,\cdots ,p$, are defined in (A2). Hence, we have

$$\begin{array}{ccc} \varphi (y) & := & -2\log L\left(\alpha_{0},\beta_{0}+\frac{y_{1}}{m},\psi_{0}+\frac{y_{2}}{\sqrt{m}}\right)+2\log L\left(\alpha_{0},\beta_{0},\psi_{0}\right)\\ & = & \xi_{n}^{⊤}\left(\beta_{0}+\frac{y_{1}}{m},\psi_{0}+\frac{y_{2}}{\sqrt{m}}\right)\Sigma^{-1}\xi_{n}\left(\beta_{0}+\frac{y_{1}}{m},\psi_{0}+\frac{y_{2}}{\sqrt{m}}\right)\\ & & -\xi_{n}^{⊤}\left(\beta_{0},\psi_{0}\right)\Sigma^{-1}\xi_{n}\left(\beta_{0},\psi_{0}\right)+o_{p}\left(1\right)\\ & = & y^{⊤}A^{⊤}\Sigma^{-1}Ay+2y^{⊤}A^{⊤}\Sigma^{-1}\xi_{n}\left(\beta_{0},\psi_{0}\right)+o_{p}\left(1\right), \end{array}$$

uniformly for βψ∈B. Then, the minimizer of φ(y) satisfies

$$\begin{array}{c} \hat{y}=-{\left(A^{⊤}\Sigma^{-1}A\right)}^{-1}A^{⊤}\Sigma^{-1}\xi_{n}\left(\beta_{0},\psi_{0}\right)+o_{p}\left(1\right). \end{array}$$

Using this, we further have

$$\begin{array}{ccc} & & -2\log L\left(\alpha_{0},\beta_{0}+\frac{{\hat{y}}_{1}}{m},\psi_{0}+\frac{{\hat{y}}_{2}}{\sqrt{m}}\right)\\ & = & \xi_{n}{\left(\beta_{0},\psi_{0}\right)}^{⊤}\left(\Sigma^{-1}-\Sigma^{-1}A{\left(A^{⊤}\Sigma^{-1}A\right)}^{-1}A^{⊤}\Sigma^{-1}\right)\xi_{n}\left(\beta_{0},\psi_{0}\right)+o_{p}\left(1\right)\\ & = & \xi_{n}{\left(\beta_{0},\psi_{0}\right)}^{⊤}{\left(\mathrm{diag}\left\{0,{\bar{\sigma }}^{2},0,\cdots ,0\right\}\right)}^{-1}\xi_{n}\left(\beta_{0},\psi_{0}\right\}+o_{p}\left(1\right)\\ & = & {\bar{\sigma }}^{-2}{\left(\frac{1}{\sqrt{m}}\sum_{t=1}^{m}Z_{t,2}\left(\alpha_{0},\beta_{0},\psi_{0}\right)\right)}^{2}+o_{p}\left(1\right)\\ & \overset{d}{⟶} & \chi_{1}^{2}, \end{array}$$

based on Lemmas 1 and 2 as n→∞. This completes the proof of this Theorem. □

**Proof of Theorem** **2.** We only show **Case (i)** as the other two cases can be proved in a similar fashion. Under the local alternative hypothesis $H_{1}$, we have

$$\begin{array}{c} Z_{t}\left(\alpha_{0},\beta ,\psi \right)=Z_{t}\left(\alpha_{0}-\frac{C_{0}}{\sqrt{m}},\beta ,\psi \right)+\frac{C_{0}}{\sqrt{m}}{\overset{˘}{X}}_{t-1}, \end{array}$$

where ${\overset{˘}{X}}_{t-1}={\left(1,w_{t+m},\Delta X_{t-1},\cdots ,\Delta X_{t-p}\right)}^{⊤}$. Then, it is easy to show as n→∞ that

(A6) $$\begin{array}{ccc} & & \frac{1}{\sqrt{m}}\sum_{t=1}^{m}Z_{t}\left(\alpha_{0},\beta ,\psi \right)\\ & = & \frac{1}{\sqrt{m}}\sum_{t=1}^{m}Z_{t}\left(\alpha_{0}-\frac{C_{0}}{\sqrt{m}},\beta ,\psi \right)+\frac{C_{0}}{\sqrt{m}}\frac{1}{\sqrt{m}}\sum_{t=1}^{m}{\overset{˘}{X}}_{t-1}\\ & = & \frac{1}{\sqrt{m}}\sum_{t=1}^{m}Z_{t}\left(\alpha_{0}-\frac{C_{0}}{\sqrt{m}},\beta ,\psi \right)+C_{0}\gamma_{1}+o_{p}\left(1\right), \end{array}$$

and

(A7) $$\begin{array}{ccc} & & \frac{1}{m}\sum_{t=1}^{m}Z_{t}\left(\alpha_{0},\beta ,\psi \right)Z_{t}{\left(\alpha_{0},\beta ,\psi \right)}^{⊤}\\ & = & \frac{1}{m}\sum_{t=1}^{m}\left(Z_{t}\left(\alpha_{0}-\frac{C_{0}}{\sqrt{m}},\beta ,\psi \right)+\frac{C_{0}}{\sqrt{m}}{\overset{˘}{X}}_{t-1}\right){\left(Z_{t}\left(\alpha_{0}-\frac{C_{0}}{\sqrt{m}},\beta ,\psi \right)+\frac{C_{0}}{\sqrt{m}}{\overset{˘}{X}}_{t-1}\right)}^{⊤}\\ & = & \frac{1}{m}\sum_{t=1}^{m}Z_{t}\left(\alpha_{0}-\frac{C_{0}}{\sqrt{m}},\beta ,\psi \right)Z_{t}{\left(\alpha_{0}-\frac{C_{0}}{\sqrt{m}},\beta ,\psi \right)}^{⊤}+o_{p}\left(1\right). \end{array}$$

Based on the quantities $\frac{1}{\sqrt{m}}\sum_{t=1}^{m}Z_{t}\left(\alpha_{0}-\frac{C_{0}}{\sqrt{m}},\beta ,\psi \right)$ and $\frac{1}{m}\sum_{t=1}^{m}Z_{t}\left(\alpha_{0}-\frac{C_{0}}{\sqrt{m}},\beta ,\psi \right)Z_{t}{\left(\alpha_{0}-\frac{C_{0}}{\sqrt{m}},\beta ,\psi \right)}^{⊤}$ given in (A6) and (A7), respectively, the rest of the proof is similar to that of Lemmas 1 and 2 and Theorem 1. We omit the details. □
